# Integrating Pharmacy and Registry Data Strengthens Clinical Assessments of Patient Adherence

**DOI:** 10.3389/fphar.2022.869162

**Published:** 2022-03-25

**Authors:** Sarah Serhal, Carol Armour, Laurent Billot, Ines Krass, Lynne Emmerton, Bandana Saini, Sinthia Bosnic-Anticevich, Bonnie Bereznicki, Luke Bereznicki, Sana Shan, Anna Campain

**Affiliations:** ^1^ Woolcock Institute of Medical Research, Sydney, NSW, Australia; ^2^ School of Pharmacy, The University of Sydney, Sydney, NSW, Australia; ^3^ Central Sydney Area Health Service, Sydney, NSW, Australia; ^4^ The George Institute, Newtown, NSW, Australia; ^5^ Faculty of Medicine, University of New South Wales, Sydney, NSW, Australia; ^6^ Curtin Medical School, Curtin University, Perth, WA, Australia; ^7^ Tasmanian School of Medicine, Hobart, TAS, Australia; ^8^ School of Pharmacy and Pharmacology, University of Tasmania, Hobart, TAS, Australia

**Keywords:** asthma, medication adherence, data linkage, pharmacy, primary care, routinely collected data, pharmacy refill data, pharmaceutical benefits scheme

## Abstract

**Background:** Accurate clinical assessment of patient adherence using reliable and valid measures is essential in establishing the presence of adherence issues and support practices for pharmacists.

**Objective:** This investigation aims to conduct a novel assessment of patient adherence to asthma controller therapy by combining 1) patient-specific dosage data found in pharmacy dispensing data with 2) centrally collected administrative claims records, to determine the added value of using both sources of data.

**Methods:** A total of 381 clinically uncontrolled asthma patients, from 95 community pharmacies across three Australian States were recruited and provided consent for the retrieval of their claims records and pharmacy dispensing data. Patients were stratified as multiple or single pharmacy users and adherence scores were calculated *via* the proportion of days covered (PDC) method using 1) patient claims records, 2) patient pharmacy dispensing data, and 3) combined claims records and pharmacy dispensing data. Cohort and subgroup adherence estimates were then compared.

**Results:** Low levels of adherence were evident amongst the cohort irrespective of the data source used. PDC estimates based on claims records alone or combined claims records and pharmacy dispensing data were significantly higher than estimates based on pharmacy dispensing data for the total cohort (56%, 52%, 42% respectively, *p* < 0.001) and more noticeably for multiple pharmacy users (67%, 64%, 35% respectively, *p* < 0.001). PDC estimates based on combined claims records and pharmacy dispensing data were significantly lower than estimates based on claims records alone, indicating that perhaps standard daily dose is not a robust proxy for prescribed dosage to inhaled respiratory devices in adherence approximations. Poorer adherence was found amongst single pharmacy users than multiple pharmacy users when combined claims records and pharmacy dispensing data (46% compared to 64% respectively, *p* < 0.001) or claims records alone (51% compared to 67% respectively, *p* < 0.001) were compared.

**Conclusion:** Access to routine collected data increases clinical acuity over patient adherence to asthma controller medications and is a valuable resource for health care professionals. A policy of secure accessibility of such data at the patient-pharmacist or patient-GP interface may allow real-time intervention and assist in decision making across numerous therapeutic areas.

## Introduction

Suboptimal medication adherence is an intractable issue that compromises patient care. Maintaining optimal adherence is a challenge regardless of the medication or the nature of the illness (Eduardo Sabaté, 2003; Elliott et al., 2006; Yeaw et al., 2009). In recent decades, long-term medication adherence for chronic conditions has been estimated to be less than 50% (Eduardo Sabaté, 2003), with predictions of this adherence gap to widen as the global population ages (Elliott et al., 2006). Poor adherence negatively impacts a patient’s health, reduces the effectiveness of treatments, and increases financial burden on patients and the health system (Haynes et al., 2008; National Collaborating Centre for Primary, 2009; Golay, 2011; GuildLink, 2021). Thus improving medication adherence is a public health concern and may benefit population health outcomes and health economics (Golay, 2011; Marcum et al., 2017; Torres-Robles et al., 2021).

Increasingly, healthcare is becoming more digitalized and large health databases are being used within pharmacoepidemiologic cohort-based research for measuring population adherence (The Organisation for Economic Co-operation and Development, 2019; The Commonwealth Fund, 2021). Sources of routinely collected medication registry data include prescribing or dispensing data, health insurance data and national health records (Kardas et al., 2020). These registry data contain five elements: 1) the drug name, 2) strength, 3) dose, 4) quantity, and 5) date of dispensing (Schneeweiss and Avorn, 2005). National health records, including routinely collected national pharmacy claims records (henceforth referred to as claims records), are often collected for national administrative purposes and are therefore accurate, unified and complete, but may lack prescribed dosage information. When using claims records, adherence estimates are based on guideline-specified (standard) doses that may not be representative of the patient’s prescribed medication regimen.

Within community pharmacy, a unique opportunity exists to detect suboptimal adherence among patients. For example, pharmacist vigilance in monitoring medication usage could prompt pharmacist-led interventions to address patient-specific adherence barriers affecting asthma control (Chan et al., 2013) and/or can enable pharmacists to effectively triage patients to appropriate care by their clinicians. Within community pharmacy, using pharmacy dispensing data to calculate medication possession rates and coverage is clinically convenient and useful (Lehmann et al., 2014). Pharmacy dispensing data are extremely valuable as they include prescribed dosage details for each patient. However, these data report exclusively what was collected at a single pharmacy. Therefore, this measure may underestimate a patient’s adherence, particularly if patients visit multiple pharmacies for convenience, or personal, clinical or financial reasons (Sansone and Sansone, 2012). In Australia, it is estimated that approximately one quarter of patients visit multiple pharmacies for their prescription medication needs, increasing to one third for other non-prescription medicines (The Pharmacy Guild of Australia, 2018; Pearson DDL, 2021).

Asthma is an incurable chronic inflammatory condition of the airways. For most patients, consistent use of preventative therapy (controller medicines) is needed to achieve symptomatic control and better health-related quality of life and minimize future exacerbation risk (National Asthma Council Australia, 2019; Global Initiative for Asthma, 2020). Like many chronic diseases, suboptimal levels of adherence amongst adults with asthma is well documented internationally (DiSantostefano et al., 2013; Reddel et al., 2015; Hull et al., 2016; Australian Institute of Health and Welfare, 2018; Cutler et al., 2018; Amin et al., 2020). However, provision of adherence support by pharmacists has been shown to improve therapeutic outcomes (Armour et al., 2007; Chan et al., 2013; Torres-Robles et al., 2021).

Through advances in e-health technology in some countries, claims records are becoming more accessible to healthcare providers *via* patient e-health records, including within community pharmacy. Thus, in the absence of a gold standard for estimating patient adherence and assisted by the knowledge that all asthma controller medicines are recorded through claims records, there is an opportunity to utilize both pharmacy dispensing data and claims records to gain a more complete understanding of a patient’s adherence to asthma controller therapy. This will enable pharmacists to efficiently direct adherence-based interventions to those most in need.

Previous studies have attempted to expand this field and ascertain adherence patterns such as the prevalence of primary non-adherence by linking general practice prescribing and pharmacy dispensing data or pharmaceutical claims records and hospitalization data (Linnet et al., 2012; Tibble et al., 2020). To the best of our knowledge this is the first study to have access to a linked set of pharmacy dispensing data and pharmaceutical claims records for a cohort of patients. Additionally, it is the first time these data sources have been combined to create a novel measure of adherence that can be compared to traditionally used methods. This investigation aimed to conduct a novel assessment of patient adherence to asthma controller therapy by combining 1) patient-specific dosage data found in pharmacy dispensing data with 2) claims records. The overall objective was to determine if the novel measure provided a clearer indication of a patient’s medication adherence and to establish a potential framework for the use of routinely collected claims data in practice.

## Materials and Methods

This study used pharmacy dispensing data and routinely collected national pharmacy claims records relating to participants in the Pharmacy Trial Program–Asthma and Rhinitis Control (PTP-ARC) (Australian Government Department of Health, 2021; Serhal et al., 2021).

A total of 381 patients, from 95 regional, remote, and metropolitan community pharmacies in the Australian states of New South Wales (NSW), Western Australia (WA) and Tasmania were recruited between August 2018 and March 2019 (Australian Government Department of Health, 2021; Serhal et al., 2021). Patients were adults aged 18 years or older with a current diagnosis of asthma. Among other variables, the PTP-ARC measured patients’ medication adherence to asthma controller therapy in the 12 months prior to enrolment in the PTP-ARC, whereupon their asthma was assessed as poorly controlled in accordance with the Asthma Control Questionnaire (ACQ score of 1.5 or over) (Juniper et al., 1999; Juniper et al., 2006).

The trial was approved by the Human Research Ethics Committees of The University of Sydney, Curtin University and The University of Tasmania, funded by the Australian Government Department of Health (Australian New Zealand Clinical Trials Registry, 2018) and registered within the Australian New Zealand Clinical Trials Registry (Registration Number ACTRN12618000313235) (Australian Government Department of Health, 2021). All participating patients provided informed consent to participate in the study and for retrieval of their medication collection records.

### Data Sources

This study uses two data sources including 1) claims records and 2) pharmacy dispensing data.

(1) Claims records are routinely collected administrative data obtained by the Australian government as part of their subsidization scheme for prescription medicines known as the Pharmaceutical Benefits Scheme (PBS) (Australian Government Department of Health, 2021). Claims records are a national data source and all medication dispensed through the PBS, within an Australian pharmacy, are recorded in a central database upon submission for reimbursement and can be linked to a patient *via* their unique Medicare ID (Australian Government Department of Health, 2021). PBS medicines are subject to a patient co-payment to a threshold amount based on patient concessional status. This dataset includes medicines both below and above this threshold (excluding items dispensed as “private” or those not on the PBS List). Separate consent was requested for collection of patient pharmaceutical claims records. Services Australia (formerly the Department of Human Services) is acknowledged for supplying the PBS information.
(2) Pharmacy dispensing data are records of all medications collected by patients from a particular pharmacy. This data is specific to the pharmacy site in which the medications were collected and are kept locally to form part of a patients records and for legal and reimbursement purposes.

All data collected for the purposes of this investigation were deidentified.

Although these data sources are similar, key differences are present in both coverage (national vs. individual pharmacies) and the presence of prescribed dosage information supplied by the treating clinician. These differences are summarized in Table 1.

**TABLE 1:** Contents, strengths, and limitations of medication data sources utilized.

Data source	Contents	Strengths	Limitations
*Claims Records*	Date of medication prescribing	Complete record of all PBS^a^ subsidized medicines, within a set time frame, that have been collected by patients from all pharmacies in Australia	Prescribed dosage not included. Only includes supplied medications with no record of unfilled prescriptions
	Date of medication supply		
	PBS^a^ item code		
	Medication name		
	Medication strength		
	Quantity supplied		
	Drug formulation		
*Pharmacy dispensing data*	Date of medication supply	Records all medicines collected, within a set time frame, by patients from a particular pharmacy including the prescribed dosage instructions	Site specific. Prescriptions collected from other pharmacies are not recorded. Only includes supplied medications with no record of unfilled prescriptions
	PBS^a^ item code		
	Medication name		
	Medication strength		
	Quantity supplied		
	Drug formulation		
	Prescribed dosage		
	Prescriber details		

a Notes: The Pharmaceutical Benefits Scheme (PBS) is an Australian Government initiative that subsidizes prescription medicines for Australian residents (Australian Government Department of Health, 2021). Any medication dispensed through the PBS, is recorded in a central database upon submission for reimbursement and can be linked to a patient *via* their unique Medicare ID. PBS medicines are subject to a patient co-payment to a threshold amount based on patient concessional status. This dataset includes medicines both below and above this threshold (excluding items dispensed as “private” or those not on the PBS List).

### Calculating Adherence

Adherence scores were calculated for each patient using the proportion of days covered (PDC). This measure refers to the proportion of days that a patient would have access to medicines based on the amount of medication dispensed, and is a measure between 0 and 100% (Raebel et al., 2013; National Center for Chronic Disease Prevention and Health Promotion, 2015; American Pharmacist Association, 2020). A PDC of 80% or higher represented adherence to controller therapy, and lower than 80% as non-adherence to controller therapy (Karve et al., 2009; Raebel et al., 2013).

$$PDC\;\left(\%\right)=\left(\frac{Number\;of\;days\;with\;medication\;available}{Number\;of\;days\;in\;the\;period}\right)x\;100$$

This calculation was performed using 1) claims records, 2) pharmacy dispensing data, and 3) combined claims records and pharmacy dispensing data.

#### Adherence Calculated *via* Claims Records

A complete 12-month national pharmacy claims history was collected for all consenting patients. Number of days with medication available was based on the date of medication supply and the number of doses supplied. Standard daily dosing was assumed due to data limitations with respect to prescribed dosage. Standard dose is defined as the minimum effective dose for adults required for each formulation/product, based on recommendations provided by the Australia Medicines Handbook (Australian Medicines Handbook, 2020), Therapeutic Guidelines (Therapeutic Guidelines Limited, 2019) and the Australian Asthma Handbook (Australia NAC, 2019).

#### Adherence Calculated *via* Pharmacy Dispensing Data

A complete 12-months pharmacy dispensing history was either collected electronically or manually for each patient. Number of days with medication available was based on the date of medication supply, the number of doses supplied and the prescribed dosage. If no dosage information was provided, the last available instructions for the prescribed medicine was carried forward; if no prior instructions were provided, the standard dose was assumed. In cases where a dose range was prescribed (e.g., 1-2 puffs), the mean dosage was used in calculations.

#### Adherence Calculated Using Combined Claims Records and Pharmacy Dispensing Data

The number of days with medication available was based on the date of dispensing, the number of doses supplied and the dose instructions. The medication supply dates were based on claims records, and prescribed dosage information was extracted from pharmacy dispensing data. If no dosage information was provided, the last available instruction for the prescribed medication was carried forward. If no instructions were available, the standard dose was assumed.

The analysis spanned all the patients’ asthma controller medicines (Global Initiative for Asthma, 2020). Anatomical therapeutic chemical codes, PBS codes and standard daily doses are available in the supplementary material.

Common assumptions in the PDC calculations include: 1) the claims records were complete and accurate 2) dosage remained consistent for the medication dispensed, 3) the purchased medicine(s) was used for the person intended 4) medication coverage (i.e., the availability of the medication), was a proxy for taking the medicine, 5) in cases when a subsequent supply was granted prior to the exhaustion of a previous supply, supply was adjusted so that the prescription start date became the date after the previous refill had ended.

## Classifying Patients as Single-or Multiple-Pharmacy Users

Adherence estimates were calculated using the aforementioned three approaches for the total cohort and then for patient subgroups based on evidence of multiple or single pharmacy use. A patient was considered a multiple-pharmacy user if there was evidence of collecting their asthma controller medicines from more than one pharmacy in the trial period. Specific pharmacies could not be identified in the claims data, therefore discrepancies in pharmacy dispensing data and claims data over the 12-month period were indicative of multiple-pharmacy use. When medication was dispensed from a pharmacy not in the study, these data would be recorded in the claims data but not in the pharmacy dispensing data. Patients who collected their asthma controller medicines from only one pharmacy were considered single pharmacy users. For single pharmacy users all records in the claims data matched pharmacy dispensing data for medication and date collected.

An additional subgroup analysis was conducted to compare patient demographic factors and clinical measures between patients who had collected asthma controller medicines from a single pharmacy versus multiple pharmacies.

## Patient Characteristics

Patient demographic data included self-reported age, gender, work status, education status, smoking status, allergic rhinitis status and asthma history information including age of asthma onset, whether the patient had a lung function test and whether the patient had an asthma-related emergency presentation and/or hospital admission in the 12 months prior to the trial. Clinical measures compared included baseline asthma control as assessed *via* the ACQ (Juniper et al., 1999), quality of life *via* the Impact of Asthma on Quality of Life Questionnaire (IAQLQ) (Marks et al., 1992), and allergic rhinitis control *via* the Rhinitis Control Assessment Test (RCAT) (Schatz et al., 2010; Meltzer et al., 2013).

## Data Analysis

**FIGURE 1:**
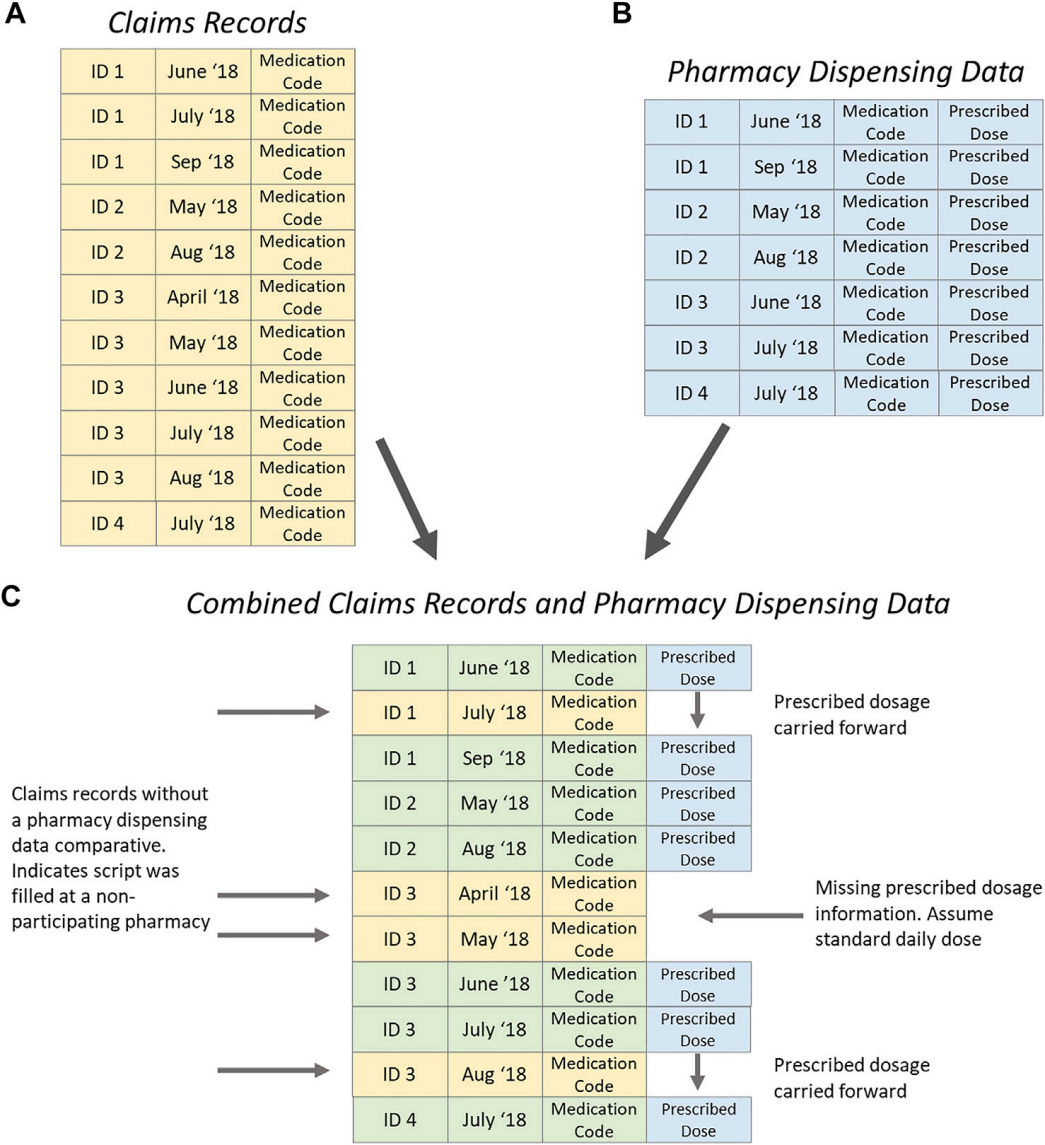
Process of merging claims records (yellow) and pharmacy dispensing data (blue) for adherence analysis.

The claims records contained all pharmaceutical claims made for each patient throughout the 12 months preceding entry to the trial (Figure 1A). Pharmacy dispensing data included all asthma controller medicines dispensed at a particular pharmacy as well as the prescribed dosage for each patient (Figure 1B). These data sources were linked by Patient (ID) and dispensing data (date). Figure 1C illustrates the data scenarios. Patient ID 1 attended multiple pharmacies, when the pharmacy was not in the study, the previously recorded prescribing dose was carried forward. Patient ID 2 attended only one pharmacy during the study, and all dosage information was available. Patient ID 3 attended multiple pharmacies; the first dispensing during the study period was not at a participating pharmacy, so no prescribed dosage was available and standard daily dose was assumed. Later in the study when Patient ID 3 attended a pharmacy not in the study, the prescribed dosage was carried forward from a previous dispensing.

The additional information obtained by including the patients’ prescribed dose in the PDC calculations was quantified by the difference between the PDC calculated *via* claims records and the PDC calculated *via* the combined claims records and pharmacy dispensing data. A secondary analysis was also performed to identify if the results achieved using PDC scores were consistent when the commonly used binary definition of adherence is used. A patient is considered adherent if their PDC score ≥80%. (Karve et al., 2009; Raebel et al., 2013) Standard summary statistics were used throughout, including measures of proportions, measures of central tendency (median and mean) and dispersion (the interquartile range (IQR) and standard deviation (SD). Absolute standardized effect sizes (SES) were used to compare groups with respect to cohort characteristics (range 0–1, higher number indicating a larger difference between the two subgroups). Effect sizes and confidence intervals as well as Student’s t-tests, both paired and unpaired, and the non-parametric Wilcoxon Rank Sum (WRS) tests were used to compare means and differences of medication adherence measures.

The analysis was performed using both SAS version 9.4, SAS Enterprise Guide 7.1 and R version 3.6.2 (R Core Team, 2018) including R packages *ggplot2* (Wickham, 2016) and *ggridges* (Wilke, 2021). All available demographic and clinical measures were used without imputation.

## Results

### Patients

Seventy-six percent (*n* = 289) of the total PTP-ARC trial cohort were included in the analysis. Fifteen percent (*n* = 57) of the total cohort were excluded as they did not collect an asthma controller medication in the 12 months preceding recruitment, while 9% (*n* = 35) did not consent to their claim’s records being accessed. Single-pharmacy users comprised 67% (*n* = 195) of the included patients.

Most patients were from NSW (72%), resided in metropolitan areas (64%), and were female (72%), 56 years of age or greater (55%), non-smokers (85%), self-reported having allergic rhinitis (74%) and self-reported a diagnosis of asthma prior to the age of 35 years (68%). All patients had poorly controlled asthma with the cohort mean ACQ score being 2.5 (Table 2).

**TABLE 2:** Baseline patient characteristics based on pharmacy use.

	Single pharmacy users (*n* = 195)	Multiple pharmacy users (*n* = 94)	Total (*n* = 289)	Absolute standardized effect size
Pharmacy state				0.277
New South Wales	133/195 (68.2%)	75/94 (79.8%)	208/289 (72.0%)	
Tasmania	23/195 (11.8%)	7/94 (7.4%)	30/289 (10.4%)	
Western Australia	39/195 (20.0%)	12/94 (12.8%)	51/289 (17.6%)	
Pharmacy remoteness^a^				0.047
Highly accessible	127/195 (65.1%)	59/94 (62.8%)	186/289 (64.4%)	
Accessible	49/195 (25.1%)	25/94 (26.6%)	74/289 (25.6%)	
Moderately accessible, remote, very remote	19/195 (9.7%)	10/94 (10.6%)	29/289 (10.0%)	
Age (years)				0.086
18–55	85/195 (43.6%)	45/94 (47.9%)	130/289 (45.0%)	
>55	110/195 (56.4%)	49/94 (52.1%)	159/289 (55.0%)	
Female	141/195 (72.3%)	68/94 (72.3%)	209/289 (72.3%)	0.001
Work Status				0.414
Full-time employed	41/195 (21.0%)	22/94 (23.4%)	63/289 (21.8%)	
Home duties	15/195 (7.7%)	11/94 (11.7%)	26/289 (9.0%)	
Part time or casually employed	48/195 (24.6%)	13/94 (13.8%)	61/289 (21.1%)	
Retired/pensioner	62/195 (31.8%)	41/94 (43.6%)	103/289 (35.6%)	
Other	29/195 (14.9%)	7/94 (7.4%)	36/289 (12.5%)	
Education				0.190
High school education or below	101/195 (51.8%)	50/94 (53.2%)	151/289 (52.2%)	
Tertiary non-university	54/195 (27.7%)	20/94 (21.3%)	74/289 (25.6%)	
University or higher	40/195 (20.5%)	24/94 (25.5%)	64/289 (22.1%)	
Self-reported age of asthma onset (years)				0.403
0–5	34/195 (17.4%)	32/94 (34.0%)	66/289 (22.8%)	
6–15	42/195 (21.5%)	17/94 (18.1%)	59/289 (20.4%)	
16–34	55/195 (28.2%)	20/94 (21.3%)	75/289 (26.0%)	
35–55	36/195 (18.5%)	15/94 (16.0%)	51/289 (17.6%)	
>55	28/195 (14.4%)	10/94 (10.6%)	38/289 (13.1%)	
Self-reported lung function test				0.173
<12 months ago	58/195 (29.7%)	26/94 (27.7%)	84/289 (29.1%)	
≥12 months ago	81/195 (41.5%)	47/94 (50.0%)	128/289 (44.3%)	
Never	56/195 (28.7%)	21/94 (22.3%)	77/289 (26.6%)	
Smoker	30/195 (15.4%)	12/94 (12.8%)	42/289 (14.5%)	0.075
Self-reported allergic rhinitis	141/195 (72.3%)	73/94 (77.7%)	214/289 (74.0%)	0.124
Emergency Department presentation in the last 12 months (Yes)	48/195 (24.6%)	28/94 (29.8%)	76/289 (26.3%)	0.116
Hospital admission in the last 12 months (Yes)	26/195 (13.3%)	22/94 (23.4%)	48/289 (16.6%)	0.262
ACQ score^b^ Median (Q1; Q3)	2.2 (1.7; 3.0)	2.2 (1.8; 3.0)	2.2 (1.7; 3.0)	0.075
IAQLQ score^c^ Median (Q1; Q3)	3.1 (1.8; 4.8)	3.1 (2.0; 5.0)	3.1 (1.8; 4.9)	0.107
RCAT score^d^ Median (Q1; Q3)	20.0 (16.0; 25.0)	21.0 (17.0; 24.0)	20.0 (16.0; 25.0)	0.098

Note: Absolute standardized differences were used to compare subgroups. Values range from 0 to 1, with a higher number indicating a larger difference between the two subgroups.

a Participating pharmacies were identified as either “highly accessible” (PhARIA Category 1), “accessible” (PhARIA Categories 2 and 3) or “moderately accessible, remote or very remote” (PhARIA Categories 4, 5 and 6) National Rural Health Alliance, 2011; The University of Adelaide, 2019a; The University of Adelaide, 2019b

b Asthma Control Questionnaire (ACQ) score lies between 0 (totally controlled) and 6 (extremely poorly controlled). A score of 1.5 or greater is considered an indication of poorly controlled asthma Juniper et al., 2006.

c Impact of Asthma on Quality of Life Questionnaire (IAQLQ) scores lie between 0 and 10. Higher scores represent a greater impact of asthma on quality of life Marks et al., 1992.

d Rhinitis Control Assessment Test (RCAT) scores lie between 6 and 30. The lower the score, the more severe the allergic rhinitis; the higher the score, the less severe the allergic rhinitis. Patients scoring ≤21 are considered clinically “symptom uncontrolled”; those scoring >21 are considered “symptom controlled“ Meltzer et al., 2013.

Table 2 presents the absolute standardized differences between multiple pharmacy users and single pharmacy users when subgroups were compared. Single-pharmacy users were comparable to multiple-pharmacy users in most characteristics; however, there were differences with respect to work status and reported age of asthma onset. A higher proportion of multiple-pharmacy users were retired or pensioners (SES = 0.414, percentage retired/pensioner 44% compared to 32%), and the reported age of asthma onset for multiple pharmacy users was younger (SES = 0.403, percentage between 0 and 5 years 34% compared to 17%) compared to single pharmacy users (Table 2).

### Adherence

The mean PDC estimate for the total cohort using pharmacy dispensing data alone was 42% (SD = 31.8%). This increased significantly to 56% (SD = 32.6%) when claims records were the only source used and to 52% (SD = 31.9%) when combining claims records and the prescribed dosage from pharmacy dispensing data (Table 3). The mean difference between the PDC calculated *via* claims records and the PDC calculated *via* the combined claims records and pharmacy dispensing data was 5%, with a standard deviation of 13.7% (Q1 = 0%, Q3 = 8.2%, *p*-value <0.001, WRS test), indicating a significant finding.

**TABLE 3:** Patient adherence.

Data source	Single-pharmacy users (*n* = 195)^a^ Mean PDC (SD)	Multiple-pharmacy users (*n* = 94)^a^ Mean PDC (SD)	Total (*n* = 289)^a^ Mean PDC (SD)	Mean difference between the PDCs for single- and multiple-pharmacy users (95% CI) (unpaired *t*-test)
Pharmacy dispensing data	45.6 (31.5)	35.2 (31.4)	42.2 (31.8)	10.4% (2.6%–18.1%) *p* = 0.009^a^
Claims records	50.7 (33.3)	67.2 (28.4)	56.1 (32.6)	16.4% (9.0%–23.9%) *p* < 0.001^a^
Combined claims records and pharmacy dispensing data	45.6 (31.5)	63.9 (29.5)	51.5 (31.9)	18.3% (10.8%–25.7%) *p* < 0.001^a^
Mean difference between PDC calculated based on pharmacy dispensing data and claims data alone (95% CI) (paired t-test)	5.1% (3.0%–7.3%) *p* < 0.001^a^	32.0% (27.1%–36.8%) *p* < 0.001^a^	13.9% (11.3%–16.4%) *p* <0.001^a^	—

a PDC refers to the Proportion of Days Covered by at least one controller medicine (Raebel et al., 2013).

Patients collecting asthma medicines from a single pharmacy had a PDC of 46% (SD = 31.5%) calculated using the pharmacy dispensing data, which increased significantly to 51% (SD = 33.3%) when using claims records alone. When these two sources were compared, the PDC estimate from claims records was equivalent to the PDC calculated using pharmacy dispensing data alone, as no additional information was gained from the claim’s records (Table 3).

Patients collecting asthma medicines from multiple pharmacies had a PDC estimate of 35% (SD = 31.4%) in analysis of pharmacy dispensing data alone, and 67% (SD = 29.5%) using claims records alone. There was a significant difference in PDC estimates between pharmacy dispensing data and the claims records of 32% (SD = 23.7%) (*p*-value < 0.001) (Table 3). When data sources were combined and adjustments to PDC were made based on the patient prescribed dose (Figure 1), the mean PDC reduced to 64% (SD = 29.5%).

Using combined data sources, single-pharmacy users were found to have a significantly lower adherence estimate than multiple-pharmacy users (18%, 95%CI 11%–26%, *p* < 0.001).

**FIGURE 2:**
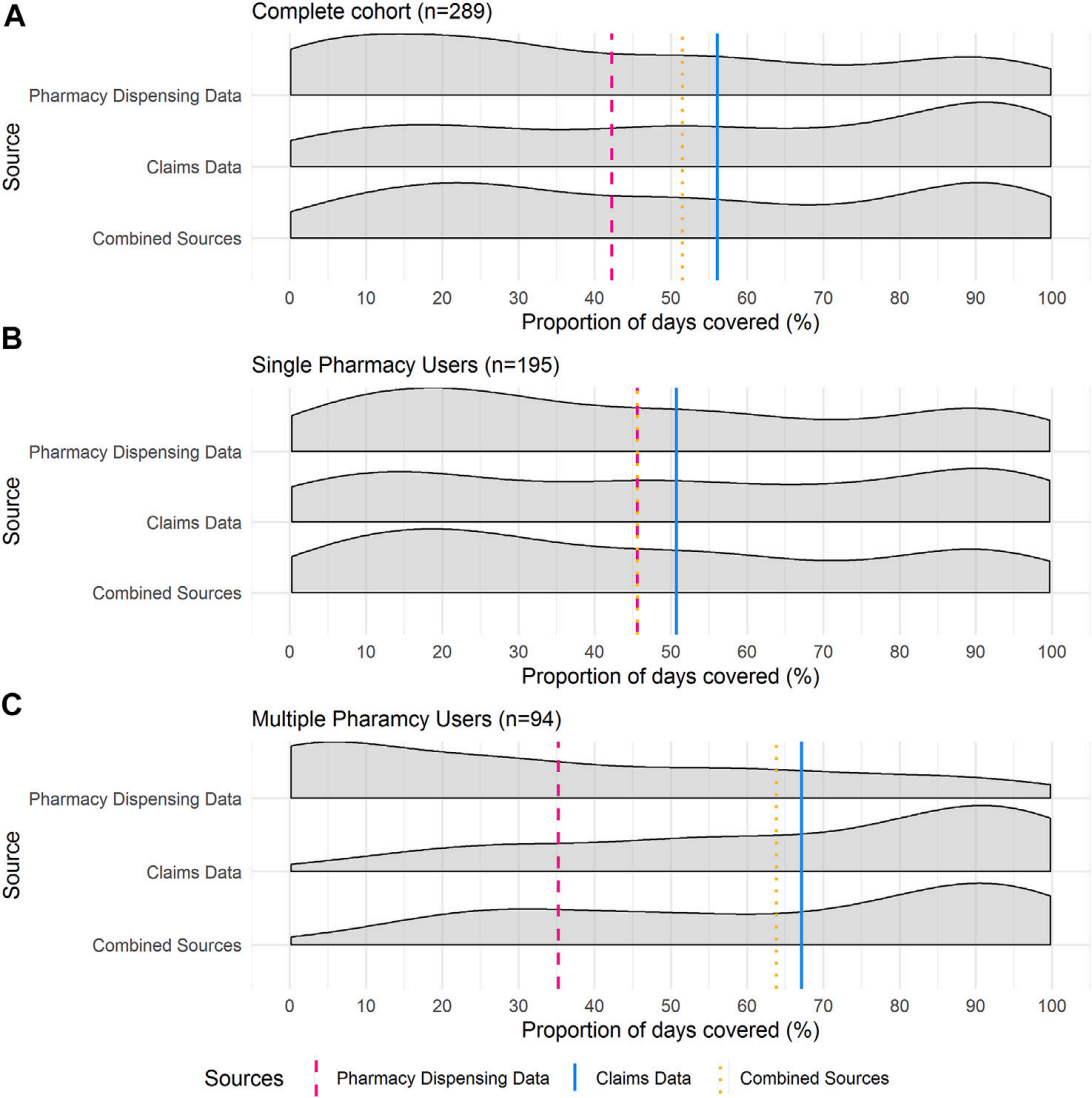
Proportion of Days Covered (PDC) Density curves for **(A)** total cohort (*n* = 289), **(B)** single-pharmacy users (*n* = 195) and **(C)** multiple-pharmacy users (*n* = 94). Vertical lines are representative of mean PDC for each data source. These distribution plots illustrate the consistently larger PDC estimates calculated *via* claims records and the relative closeness in PDC estimates between the claim’s records and the combined claims records and pharmacy dispensing data.

Density plots in Figure 2 show the distribution of the patients’ PDC for the investigated 12-month period by data source and pharmacy use. The distribution of the complete cohort is presented in Figure 2A and comprises both single- and multiple-pharmacy users.

When pharmacy dispensing data were considered, a higher proportion of single-pharmacy users had a lower PDC compared to when claims records were used to calculate PDC (Figure 2B).

The pharmacy dispensing data for multiple pharmacy users was positively skewed, with a large proportion of patients having lower PDC estimates. Conversely, the distribution of the PDC calculated by claims records was negatively skewed, with most patients having a PDC > 80% (Figure 2C).

These distributions highlight the differences in the mean PDC values based on the different data sources. They illustrate the consistently larger PDC estimates calculated *via* claims records and the relative closeness in PDC estimates between the claim’s records and the combined claims records and pharmacy dispensing data.

**FIGURE 3:**
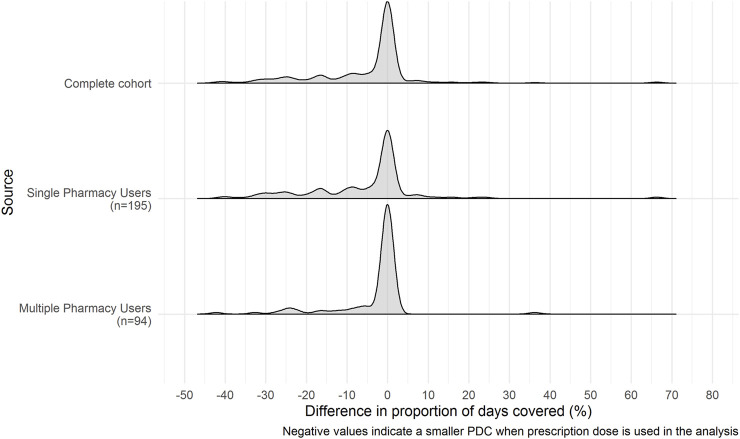
Distribution of differences in Proportion of Days Covered estimates between claims records alone and combined claims records and pharmacy dispensing data. Negative values indicate a lower PDC when patients prescribed dose is included in the analysis instead of the standard dose assumption. The differences between the PDC estimates based on patients prescribed dose versus the standard dose assumption have a skewed distribution. Therefore, it is likely that greater dose variability amongst some asthma patients within the cohort may have contributed to this finding.

The distributions of change in PDC estimates between claims records and combined claims records and pharmacy dispensing data are shown in Figure 3. All three cohorts are negatively skewed with a center around zero. This indicates that the standard daily dose assumption used when PDC estimates are calculated using claims records alone underestimate the PDC compared to when estimates are calculated using the combined claims records and pharmacy dispensing data.

For the complete cohort, the mean difference between the PDC calculated *via* claims records and the PDC calculated *via* the combined claims records and pharmacy dispensing data was −5%, (SD = 13.7) with an interquartile range of 8.2% (Q1 = −8.2%, Q3 = 0%, *p*-value < 0.001, WRS test). This difference indicates that the PDC calculated using the prescription dose information was lower than when standard dose was assumed. The standard daily dose assumption overestimated the PDC coverage by 4.6%. For single-pharmacy users, the mean change in PDC was -5% (SD = 15.3%), with an IQR of 11.1% (Q1 = −11.1%, Q3 = 0%, *p*-value < 0.001, WRS test), and for multiple pharmacy users, the mean change in PDC was −3% (SD = 9.4), with an IQR of 0.4% (Q1 = −0.4%, Q3 = 0%, *p*-value <0.001, WRS test).

All PDC findings were consistent when the sensitivity analysis was performed based on the binary measure of the proportion of patient’s adherent (PDC ≥ 80%) (see Supplementary Material).

## Discussion

A novel assessment of patient adherence to asthma controller therapy was conducted by combining patient-specific prescribed dosage data found in pharmacy dispensing data with routinely collected claims records to determine the added value of using both sources of data. PDC estimates based on pharmacy dispensing data alone or combined claims records and pharmacy dispensing data were significantly lower than estimates based on claims records alone, indicating that perhaps standard daily dose is not a robust proxy for prescribed dosage to inhaled respiratory devices in adherence approximations. However, PDC estimates based on combined pharmacy dispensing and claims records or claims records alone were significantly higher than estimates based on pharmacy dispensing data alone for the total cohort and more noticeably for multiple pharmacy users. Thus, the use of claims records over site-specific pharmacy dispensing data and the use of patient specific prescribed dosage adds value to clinical assessments and provides a clearer indication of a patient’s medication adherence.

There was a significant difference when utilizing patient-specific prescribed doses sourced from pharmacy dispensing data over the standard dose assumption. This challenges the methodology and assumptions used in prior claims-based pharmacoepidemiologic research. However, whether these differences are clinically significant in practice and reproducible in other therapeutic areas requires further research. It should be noted that the differences between the PDC estimates based on patients prescribed dose versus the standard dose assumption have a skewed distribution. Therefore, it is likely that greater dose variability amongst some asthma patients within the cohort may have contributed to this finding. Future exploration would be interesting to determine why this is the case for some patients and how these patients and their medication management differs from the majority of the cohort.

Adherence was poor amongst this cohort, irrespective of the data source, and across all subgroups. Low levels of adherence are consistent with the literature (Price et al., 2014; Price et al., 2015; Reddel et al., 2015). Moreover, the single-pharmacy users had considerably lower levels of adherence than their multiple-pharmacy user counterparts. This may seem counter intuitive and in direct contrast to available literature which supports association of multiple pharmacy use with lower medication adherence and increased risk of drug-drug interactions (Taitel et al., 2012). The difference between our investigation and those published may reflect the different therapeutic areas and medicines being investigated or international differences in the patient and pharmacy cohorts. Our results suggest that, suboptimal adherence remains a significant issue that requires addressing before a more beneficial clinical trajectory for asthma patients can be realized to reduce the associated health economic burden (Cutler et al., 2018). There is opportunity for pharmacists to improve upon this low adherence by using targeted interventions when regular patients collect medications.

Pharmacy dispensing data consistently underestimated patient adherence to therapy particularly for multiple-pharmacy users. There is a disconnect between the data that pharmacists can access and the data that can more fully inform pharmacists about a patient’s adherence. However, routinely collected claims records could complement site-specific pharmacy dispensing data and thus increase a pharmacist’s assessment of a patient’s medication adherence. This is likely to be of benefit in many therapeutic areas. Expanding the pharmacist’s access to data allows them to make clinical judgements with greater clarity and to offer better patient specific care. Furthermore, the use of claims based records in place of pharmacy-based data will improve sensitivity of adherence software programs currently used in community pharmacies to focus on patients with adherence issues (GuildLink, 2021).

The advantages of centralized and accessible registry data are apparent and recognized internationally (Wright and Twigg, 2016; Nelson et al., 2017; Jackson and Peterson, 2019; The Organisation for Economic Co-operation and Development, 2019; The Commonwealth Fund, 2021). These findings offer another clinical incentive for countries still operating with fragmented reporting networks to work towards the creation of a central data system which would be better able to serve patients and assist in real time clinical decision making. Within community pharmacy, the use of electronic health record data has the ability to elevate current standards of practice by providing a holistic view of patient management and assisting in reducing medication misadventure (Wright and Twigg, 2016; Jackson and Peterson, 2019). For example, in Australia, the increasing integration of patient electronic health records (My Health Records) (Australian government Australian Digital Health Agency, 2019) into primary care and community pharmacy allows pharmacists access to complete claims records for consenting patients under their care (Australia TPSo, 2019). However, research exploring application of these opportunities within community pharmacy practice is limited. With regard to adherence, the use of centralized data is centered on monitoring trends in medicine consumption and spending at national and cohort levels, rather than how such information could be used on a patient-by-patient basis to improve health outcomes for individuals (The Organisation for Economic Co-operation and Development, 2019; The Commonwealth Fund, 2021). Further work is needed to realize the full utility of centralized datasets in community pharmacy practice and automated systems and specific frameworks developed to facilitate this. This will allow integration with workflow and software to optimize health benefits and best safeguards patient privacy (Wright and Twigg, 2016; Kosari et al., 2020).

Our findings prompt reflection on pre/post adherence intervention-based studies using pharmacy dispensing data alone as an outcome measure (Armour et al., 2008; Taitel et al., 2012; Pringle et al., 2014; Stewart et al., 2014). Not only was there the possibility that adherence may have been underestimated, limited by data available at the time, it would also be difficult to differentiate between improved adherence based on the intervention in question and improved loyalty to a pharmacy, or confounding between these factors and a patient’s adherence. Collection of medicines from a single pharmacy providing a better quality of care would improve the apparent adherence estimate over time compared to where a patient continued to collect medications from multiple pharmacies based on convenience.

Allowing access to routinely collected data may also benefit general practitioners. Within general practice, knowledge of a patient’s adherence can assist by breaking the cycle of uncontrolled asthma symptoms, review and therapy escalation that ensues if suboptimal adherence is left undetected (Serhal et al., 2020). Clinicians would be able to differentiate poor asthma control as a result of suboptimal adherence from poor therapeutic response to medicines. The utility of marrying two data sources would also prove useful within a general practice setting. Prescribing data combined with claims records would overcome practitioner limitations when it comes to monitoring for primary non-adherence: whether a patient is having their prescribed medicines dispensed (Tibble et al., 2020) or “doctor shopping” practices that could lead to the overestimation or underestimation of a patient’s adherence. This methodology could also be applied to other therapeutic areas in practice and in future research to enrich patient chronic care management and offer positive implications for drugs of addiction or abuse potential i.e., real time monitoring of patient opioid use and oversight of doctor and pharmacy shopping practices.

In the future, there could be benefit in a simple multiplication factor being created *via* analysis of claims records and used as clinical tool for pharmacists to approximate patient adherence based on pharmacy data. However, this would require repeated investigations and validation, and may differ depending on the therapeutic area.

### Strengths and Limitations

To the best of our knowledge this is the first study to have access to a linked set of pharmacy dispensing data and pharmaceutical claims records for a cohort of patients. Additionally, it is the first time these data sources have been combined to create a novel measure of adherence that can be compared to traditionally used methods.

Measures of adherence disclosed in this manuscript are proxy measures of adherence. These measures represent medicine acquisition, but not necessarily medicine usage.

Adherence estimates were based on any asthma controller medicines collected within a set period, which assumes patients had not changed their behaviors prior to or during the study, i.e., there was no stockpiling of medicines by patients. However, the same rule applied to both data sources, and as this study focuses on comparing adherence rates and not the rates themselves, it is expected this effect would have minimal impact on the findings.

Thirty-three percent of patients collected their asthma medications from multiple pharmacies, despite an inclusion criterion that patients should be regular patrons of the pharmacy in which they were recruited. Despite this anomaly, this 33% figure is consistent with available literature (Look and Mott, 2003; Marcum et al., 2014; Marcum et al., 2017; The Pharmacy Guild of Australia, 2018; Pearson DDL, 2021).

## Conclusion

Access to routinely collected claims records and patient prescribed dosage increases clinical acuity of patient adherence estimates to asthma controller medicines and is a valuable resource for healthcare professionals. Secure accessibility of such data at the patient-pharmacist or patient-GP interface may allow real-time intervention and assist in decision making across numerous therapeutic areas.

## Data Availability

The datasets presented in this article are not readily available because Pharmaceutical Benefits Scheme data is subject to approval by Services Australia prior to distribution. Requests to access the datasets should be directed to sarah.serhal@sydney.edu.au.

## Abbreviations

SES, Absolute standardized effect sizes; ACQ, Asthma control questionnaire; IAQLQ, Impact of asthma on quality-of-life questionnaire; IQR, Interquartile range; PBS, Pharmaceutical Benefits Scheme; PTP-ARC, Pharmacy Trial Program—Asthma and Rhinitis Control; PDC, Proportion of days covered; RCAT, Rhinitis control assessment test; SD, Standard deviation; WRS, Wilcoxon Rank Sum.
