# Supplementary material for: Integrating Pharmacy and Registry Data Strengthens Clinical Assessments of Patient Adherence
Source: Front Pharmacol. 2022 Mar 25;13:869162. doi: 10.3389/fphar.2022.869162 (PMC8990834; doi:10.3389/fphar.2022.869162)
Supplement: Supplementary file 1 [file DataSheet1.docx]

Supplementary Material

Supplementary Table 1. Anatomical therapeutic chemical codes, PBS codes and standard daily doses

| **PBS code** | **ATC** | **Medication Class** | **Medication Name** | **Device Type** | **Strength** | **Pack Size** | **Standard Dosage** |
| --- | --- | --- | --- | --- | --- | --- | --- |
| **08141L** | **R03A** | LABA | *Salmeterol* | *Accuhaler* | 50 mcg | 60 | 1 bd (1 twice a day) |
| **08239P** | **R03A** | LABA | *Formoterol Fumarate Dihydrate* | *Turbuhaler* | 6 mcg | 60 | 1 bd (1 twice a day) |
| **08240Q** | **R03A** | LABA | *Formoterol Fumarate Dihydrate* | *Turbuhaler* | 12 mcg | 60 | 1 bd (1 twice a day) |
| **08136F** | **R03A** | LABA | *Formoterol Fumarate Dihydrate* | *Handihaler and tablets* | 12 mcg | 60 | 1 bd (1 twice a day) |
| **Private** | **R03D** | LTRA | *Montelukast* | *Chewable Tablets* | 10mg | 28 | 1 d (1 daily) |
| **08628D** | **R03D** | LTRA | *Montelukast* | *Chewable Tablets* | 5mg | 28 | 1 d (1 daily) |
| **08627C** | **R03D** | LTRA | *Montelukast* | *Chewable Tablets* | 4mg | 28 | 1 d (1 daily) |
| **08767K** | **R01A** | Cromones (Mast Cell stabilisers) | *Sodium Cromoglycate* | *Metered Dose Inhaler* | 1 mg | 200 | 10 puffs 3-4 times per day |
| **08334P** | **R01A** | Cromones (Mast Cell stabilisers) | *Sodium Cromoglycate* | *Metered Dose Inhaler* | 5 mg | 112 | 2 qid (2 four times or day) |
| **08365G** | **R01A** | Cromones (Mast Cell stabilisers) | *Nedocromil Sodium* | *Metered Dose Inhaler* | 2 mg | 112 | 2 bd (2 twice daily) |
| **08516F** | **R03B** | ICS | *Fluticasone Propionate* | *Metered Dose Inhaler* | 50 mcg | 120 | 2 bd (2 twice daily) |
| **08345F** | **R03B** | ICS | *Fluticasone Propionate* | *Metered Dose Inhaler* | 125 mcg | 120 | 1 bd (1 twice daily) |
| **08346G** | **R03B** | ICS | *Fluticasone Propionate* | *Metered Dose Inhaler* | 250 mcg | 120 | 1 bd (1 twice daily) |
| **08147T** | **R03B** | ICS | *Fluticasone Propionate* | *Accuhaler* | 100 mcg | 60 | 1 bd (1 twice a day) |
| **08148W** | **R03B** | ICS | *Fluticasone Propionate* | *Accuhaler* | 250 mcg | 60 | 1 bd (1 twice a day) |
| **08149X** | **R03B** | ICS | *Fluticasone Propionate* | *Accuhaler* | 500 mcg | 60 | 1 bd (1 twice a day) |
| **08853Y** | **R03B** | ICS | *Ciclesonide* | *Metered Dose Inhaler* | 80 mcg | 120 | 1 d (1 daily) |
| **08854B** | **R03B** | ICS | *Ciclesonide* | *Metered Dose Inhaler* | 160 mcg | 120 | 1 d (1 daily) |
| **02070Y** | **R03B** | ICS | *Budesonide* | *Turbuhaler* | 100 mcg | 200 | 1 bd (1 twice a day) |
| **02071B** | **R03B** | ICS | *Budesonide* | *Turbuhaler* | 200 mcg | 200 | 1 bd (1 twice a day) |
| **02072C** | **R03B** | ICS | *Budesonide* | *Turbuhaler* | 400 mcg | 200 | 1 bd (1 twice a day) |
| **08406K** | **R03B** | ICS | *Beclometasone Dipropionate* | *Metered Dose Inhaler* | 50 mcg | 200 | 1 bd (1 twice a day) |
| **08407L** | **R03B** | ICS | *Beclometasone Dipropionate* | *Metered Dose Inhaler* | 100 mcg | 200 | 1 bd (1 twice a day) |
| **08408M** | **R03B** | ICS | *Beclometasone Dipropionate* | *Autohaler* | 50 mcg | 200 | 1 bd (1 twice a day) |
| **08409N** | **R03B** | ICS | *Beclometasone Dipropionate* | *Autohaler* | 100 mcg | 200 | 1 bd (1 twice a day) |
| **08517G** | **R03A** | ICS + LABA | *Fluticasone Propionate/ Salmeterol* | *Metered Dose Inhaler* | 50 mcg/25 mcg | 120 | 2 bd (2 twice daily) |
| **08518H** | **R03A** | ICS + LABA | *Fluticasone Propionate/ Salmeterol* | *Metered Dose Inhaler* | 125 mcg/25 mcg | 120 | 1 bd (1 twice daily) |
| **08519J** | **R03A** | ICS + LABA | *Fluticasone Propionate/ Salmeterol* | *Metered Dose Inhaler* | 250 mcg/25 mcg | 120 | 1 bd (1 twice daily) |
| **08430Q** | **R03A** | ICS + LABA | *Fluticasone Propionate/ Salmeterol* | *Accuhaler* | 100 mcg/50 mcg | 60 | 1 bd (1 twice a day) |
| **08431R** | **R03A** | ICS + LABA | *Fluticasone Propionate/ Salmeterol* | *Accuhaler* | 250 mcg/50 mcg | 60 | 1 bd (1 twice a day) |
| **08432T** | **R03A** | ICS + LABA | *Fluticasone Propionate/ Salmeterol* | *Accuhaler* | 500 mcg/50 mcg | 60 | 1 bd (1 twice a day) |
| **08796Y** | **R03A** | ICS + LABA | *Budesonide/Formoterol Fumarate Dihydrate* | *Turbuhaler* | 100 mcg/6 mcg | 120 | 1 bd (1 twice a day) |
| **12101X** |  | ICS + LABA | *Budesonide/Formoterol Fumarate Dihydrate* | *Turbuhaler* | 100 mcg/6 mcg | 120 | 1 bd (1 twice a day) |
| **08625Y** | **R03A** | ICS + LABA | *Budesonide/Formoterol Fumarate Dihydrate* | *Turbuhaler* | 200 mcg/6 mcg | 120 | 1 bd (1 twice a day) |
| **12041R** | **R03A** | ICS + LABA | *Budesonide/Formoterol Fumarate Dihydrate* | *Turbuhaler* | 200 mcg/6 mcg | 120 | 1 bd (1 twice a day) |
| **12093L** | **R03A** | ICS + LABA | *Budesonide/Formoterol Fumarate Dihydrate* | *Turbuhaler* | 200 mcg/6 mcg | 120 | 1 bd (1 twice a day) |
| **08750M** | **R03A** | ICS + LABA | *Budesonide/Formoterol Fumarate Dihydrate* | *Turbuhaler* | 400 mcg/12 mcg | 120 | 1 bd (1 twice a day) |
| **10024N** | **R03A** | ICS + LABA | *Budesonide/Formoterol Fumarate Dihydrate* | *Rapihaler* | 50 mcg/3 mcg | 240 | 2 bd (2 twice daily) |
| **12100W** |  | ICS + LABA | *Budesonide/Formoterol Fumarate Dihydrate* | *Rapihaler* | 50 mcg/3 mcg | 240 | 2 bd (2 twice daily) |
| **10015D** | **R03A** | ICS + LABA | *Budesonide/Formoterol Fumarate Dihydrate* | *Rapihaler* | 100 mcg/3 mcg | 240 | 1 bd (1 twice a day) |
| **12042T** | **R03A** | ICS + LABA | *Budesonide/Formoterol Fumarate Dihydrate* | *Rapihaler* | 100 mcg/3 mcg | 240 | 1 bd (1 twice a day) |
| **12089G** | **R03A** | ICS + LABA | *Budesonide/Formoterol Fumarate Dihydrate* | *Rapihaler* | 100 mcg/3 mcg | 240 | 1 bd (1 twice a day) |
| **10018G** | **R03A** | ICS + LABA | *Budesonide/Formoterol Fumarate Dihydrate* | *Rapihaler* | 200 mcg/6 mcg | 240 | 1 bd (1 twice a day) |
| **12082X** | **R03A** | ICS + LABA | *Budesonide/Formoterol Fumarate Dihydrate* | *Rapihaler* | 200 mcg/6 mcg | 240 | 1 bd (1 twice a day) |
| **11273H** |  | ICS + LABA | *Budesonide + Formoterol (eformoterol)* | *Spiromax* | 200 mcg/ 6 mcg | 120 | 1 bd (1 twice a day) |
| **12029D** | **R03A** | ICS + LABA | *Budesonide + Formoterol (eformoterol)* | *Spiromax* | 200 mcg/ 6 mcg | 120 | 1 bd (1 twice a day) |
| **11301T** | **R03A** | ICS + LABA | *Budesonide + Formoterol (eformoterol)* | *Spiromax* | 400 mcg/12 mcg | 120 | 1 bd (1 twice a day) |
| **02827T** | **R03A** | ICS + LABA | *Fluticasone Propionate/Formoterol Fumarate Dihydrate* | *Metered Dose Inhaler* | 50 mcg/5 mcg | 120 | 2 bd (2 twice daily) |
| **10007Q** | **R03A** | ICS + LABA | *Fluticasone Propionate/Formoterol Fumarate Dihydrate* | *Metered Dose Inhaler* | 125 mcg/5 mcg | 120 | 1 bd (1 twice daily) |
| **10008R** | **R03A** | ICS + LABA | *Fluticasone Propionate/Formoterol Fumarate Dihydrate* | *Metered Dose Inhaler* | 250 mcg/10 mcg | 120 | 1 bd (1 twice daily) |
| **11124L** | **R03A** | ICS + LABA | *Fluticasone Furoate/Vilanterol* | *Ellipta* | 100 mcg/25 mcg | 30 | 1 d (1 daily) |
| **11129R** | **R03A** | ICS + LABA | *Fluticasone Furoate/Vilanterol* | *Ellipta* | 200 mcg/25 mcg | 30 | 1 d (1 daily) |
| **11719T** | **R03B** | ICS | *Fluticasone Furoate* | *Ellipta* | 100 mcg/25 mcg | 30 | 1 d (1 daily) |
| **11729H** | **R03B** | ICS | *Fluticasone Furoate* | *Ellipta* | 200 mcg/25 mcg | 30 | 1 d (1 daily) |
| **08230E** | **R03D** | *Theophylline* | *Theophylline* | *Oral Tablet* | 200mg | 100 | 3 d (3 daily) |
| **02634P** | **R03D** | *Theophylline* | *Theophylline* | *Oral Tablet* | 250mg | 100 | 2 d (2 daily) |
| **08231F** | **R03D** | *Theophylline* | *Theophylline* | *Oral Tablet* | 300mg | 100 | 2 d (2 daily) |
| **02614N** | **R03D** | *Theophylline* | *Theophylline* | *Oral Liquid* | 133.33mg/25mL | 500ml |  |

Supplementary Table 2. Dichotomized adherence results based on proportion of patient’s adherent

|  | **Single-pharmacy users**   (n=195) | **Multiple-pharmacy users**  (n=94) | **Total**  (n=289) |
| --- | --- | --- | --- |
| **Pharmacy dispensing data**  *Proportion of adherent patients  (PDC^1^ ≥ 80%)* | 46/195 (23.6%) | 19/94 (12.8%) | 58/289 (20.1%) |
| **Claims records**  *Proportion of adherent patients*  *(PDC^1^ ≥ 80%)* | 55/195 (28.2%) | 43/94 (45.7%) | 98/289 (33.9%) |
| **Combined claims records and pharmacy dispensing data**  *Proportion of adherent patients*  *(PDC^1^ ≥ 80%)* | 46/195 (23.6%) | 40/94 (42.6%) | 86/289 (29.8%) |

*Notes:*

1. **PDC refers to the Proportion of Days Covered by at least one asthma controller medicine^31^*

***Patients excluded:***

- *Patient did not consent to claims record collection (n=35).*
- *Patient did not collect an asthma preventer medication over the study duration (n=57)*

**Supplementary Figure 1.** Patient inclusion and stratification in analysis. *A patient was considered a multiple-pharmacy user if there was evidence of the patient collecting their asthma controller medicines from more than one pharmacy in the 12 months preceding recruitment. Patients who collected their asthma controller medicines from only one pharmacy were considered single pharmacy users.*
